# A Case Where the Vaccine Inoculation Was Supposed to Have Failed

**Published:** 1802-12-01

**Authors:** 


### *A Case where the Vaccine Inoculation was supposed to have failed; communicated by* Mr. Pears.

In reply to the obſervations of Mr. Ring, which I have been prevented from noticing ſooner (excepting in a private converſation with himſelf) I would beg leave to obſerve that the ſtatement was there given as that of the mother, (whom Mr. R. "believes to be fully entitled to credit," though he ſays erroneous in the particular to which he alludes) a method which I have always obſerved, and ſo expreſſed, to prevent any imputation upon another perſon that might appear unjuſt. Moreover, upon my afterwards aſking Mr. H. if he thought my ſtatement in the Journal was correct, he ſaid, "It was ſo."

It appears to me ſo important to bring forward all ſuch caſes as ſeem to be inimical to the *Cow-pock* inoculation, that I think it the only way to eſtabliſh the truth. I am the more anxious on this ſubject, from the aſſurance that all the caſes of ſuppoſed failures, of which I have heard, had in a degree produced effects injurious to the Vaccine Inoculation; and when they are ſuffered to remain in ſecret, or to die away, as it has been called, it affords a victory to the enemies of the vaccine practice, while the abettors of it have ſuch caſes conſtantly treaſured up, and oppoſed to them as unanſwerable, becauſe uninveſtigated, and therefore unrefuted.

On this account I ſhall trouble you with another caſe of the ſame kind, the reſult of which I am happy to find upon enquiry, proves equally favorable to the Cow-pock with the reſt; and as ſuch, ſhall be detailed for the information of thoſe who either had not, or did not, improve the opportunity for its examination, if you think proper to give it a place.

Henry Moggeridge, near five years old, was inoculated for Cow-pock, at the Small-pox Hoſpital, about twelve months ſince, and underwent its ſeveral ſtages in the uſual way. About five months ſince, he was permitted to be with a child labouring under Small-pox, of which it died, and with whom *he slept every night for two weeks previous to its death, without receiving any infection.* In the beginning of Auguſt, an eruption appeared upon the above child (Henry), which upon the inſpection of a ſurgeon was pronounced to be Small-pox, and as ſuch it was reported throughout the neighbourhood of the child, and produced conſiderable influence upon the minds of many perſons, injurious to the Cow-pock Inoculation. The ſame report and influence was extended alſo for ſome miles, in which way it came to my knowledge. The ſpots of this eruption were large, equalling the ſize of a ſixpence, and ſome of them that of a ſhilling,

ſhilling; they filled with a limpid fluid, which afterwards maturated, and in about three weeks went off, the cruſts or heads remaining two weeks afterwards; the ſcars when I ſaw them being left, and having the appearance of large and irregular ſpots, from their union with each other, eſpecially upon the back, where were the greateſt number of them, although they exiſted in all other parts of the body, and on the ſcalp. They gave me the idea of its having been what is called "*Swine Pock.*"

The child is perfectly well, and his parents entirely convinced that the Cow-pock is not at all influenced by this circumſtance, more eſpecially, as it was ſo ſtrongly confirmed by that of the child *sleeping for a fortnight with another child who died of Small-pox, without receiving the infection.*

*Rockingham Row, Newington Butts, Oct.* 20, 1802.

